# How to practice person‐centred care: A conceptual framework

**DOI:** 10.1111/hex.12640

**Published:** 2017-11-19

**Authors:** Maria J. Santana, Kimberly Manalili, Rachel J. Jolley, Sandra Zelinsky, Hude Quan, Mingshan Lu

**Affiliations:** ^1^ Department of Community Health Sciences Cumming School of Medicine University of Calgary Calgary AB Canada; ^2^ Patient Partner Strategy for Patient‐oriented Research, Methods and Development Platform Alberta AB Canada; ^3^ Department of Economics University of Calgary Calgary AB Canada

**Keywords:** conceptual framework, healthcare quality, implementation, person‐centred care

## Abstract

**Background:**

Globally, health‐care systems and organizations are looking to improve health system performance through the implementation of a person‐centred care (PCC) model. While numerous conceptual frameworks for PCC exist, a gap remains in practical guidance on PCC implementation.

**Methods:**

Based on a narrative review of the PCC literature, a generic conceptual framework was developed in collaboration with a patient partner, which synthesizes evidence, recommendations and best practice from existing frameworks and implementation case studies. The Donabedian model for health‐care improvement was used to classify PCC domains into the categories of “Structure,” “Process” and “Outcome” for health‐care quality improvement.

**Discussion:**

The framework emphasizes the structural domain, which relates to the health‐care system or context in which care is delivered, providing the foundation for PCC, and influencing the processes and outcomes of care. Structural domains identified include: the creation of a PCC culture across the continuum of care; co‐designing educational programs, as well as health promotion and prevention programs with patients; providing a supportive and accommodating environment; and developing and integrating structures to support health information technology and to measure and monitor PCC performance. Process domains describe the importance of cultivating communication and respectful and compassionate care; engaging patients in managing their care; and integration of care. Outcome domains identified include: access to care and Patient‐Reported Outcomes.

**Conclusion:**

This conceptual framework provides a step‐wise roadmap to guide health‐care systems and organizations in the provision PCC across various health‐care sectors.

## 1 INTRODUCTION

In the Institute of Medicine's 2001 seminal report *Crossing the Quality Chasm*, patient‐centred care was identified as an essential foundation for health‐care quality and patient safety^1^ and ever since has been recognized as a high priority for the delivery of health‐care services in many jurisdictions.^2^, ^3^, ^4^, ^5^, ^6^

Patient‐centred care has been an evolving concept, originally depicted by Edith Balint in 1969 as “understanding the patient as a unique human being.”^7^ Since then, there have been many other conceptualizations of patient‐centred care.^1^, ^8^, ^9^, ^10^, ^11^ Patient‐centred care has been described through an array of alternative and more commonly adopted terms, including: patient (and family)–centred care, relationship‐centred care, personalized care and user/client‐centred care. Various jurisdictions, organizations and health‐care systems utilize different terms and concepts. For instance, in the United States, the concept is usually linked to a “patient‐centred care medical model,” while in the United Kingdom, it is associated with primary care, and in Scotland, PCC is known as “mutuality.”^8^ Given that the concept of patient‐centred care is evolving, it is important to understand how different jurisdictions define and operationalize it. In this article, we have chosen to use the term “person‐centred care” (referred to as PCC), as opposed to patient‐centred care, in agreement with Ekman et al*'s* distinction between patient‐centred care and PCC, by which PCC refrains from reducing the person to just their symptoms and/or disease.^12^ We concur that it is important to acknowledge the notion of person, which calls for a more holistic approach to care that incorporates the various dimensions to whole well‐being, including a person's context and individual expression, preferences and beliefs.^12^ Additionally, PCC is not limited to only the patient, but also includes families and caregivers who are involved, those who are not living with illness, as well as prevention and promotion activities.

PCC has not been traditionally integrated into health‐care quality improvement. Recent policies emphasize the value of patient views, which not only complement health‐care provider perspectives, but also provide unique information about health‐care effectiveness,^13^, ^14^, ^15^, ^16^, ^17^, ^18^ including improvement of patient experiences and outcomes and health‐care provider satisfaction, while decreasing health‐care services utilization and costs.^19^, ^20^ Based on this evidence and the need to address sky‐rocketing health‐care costs, many health‐care systems around the world are moving towards a PCC model.^21^, ^22^, ^23^ At the global level, the World Health Organization (WHO) has developed policy frameworks for people‐centred health care^24^ highlighting person‐centredness as a core competency of health workers,^25^ and as a key component of health‐care quality^26^ and primary care.^27^

Conceptually, PCC is a model in which health‐care providers are encouraged to partner with patients to co‐design and deliver *personalized* care that provides people with the high‐quality care they need and improve health‐care system efficiency and effectiveness.

Despite many efforts to practice PCC, most health‐care systems are challenged by effective implementation of PCC across the continuum of care. Shifting to PCC requires services and roles to be re‐designed and re‐structured to be more conducive to a PCC model. Although numerous conceptual frameworks of PCC have been introduced and discussed in the existing literature,^5^, ^9^, ^11^, ^12^, ^28^, ^29^, ^30^, ^31^, ^32^, ^33^, ^34^, ^35^, ^36^, ^37^, ^38^ practical guidance on the implementation of PCC has not been well described. To address this gap, we developed a conceptual PCC framework that provides a comprehensive perspective, particularly with respect to the foundations needed to achieve PCC.

## 2 METHODS

The guiding perspective for developing the framework was from a patient (and family caregiver, representative) perspective to ensure that the framework reflects what matters people, not only policy makers and HCPs. This conceptual framework describes and links key PCC domains and best practices to a model of practical implementation, through a narrative overview^39^ of theoretical and conceptual works from academic and grey literature, in addition to policy and organizational documents.

### 2.1 Sources of information

Based on the guidance from Green et al on conducting a narrative review,^39^ a preliminary search was conducted. A number of sources included in the review were identified through a scoping review conducted on person‐centred quality indicators that revealed rich literature on PCC practice and measurement. Search protocol details, including databases and search terms, have been published.^40^ Additional works that were hand‐searched and selected included frequently cited PCC literature and key policy documents from reference lists, and those identified by our patient partner (Zelinsky).

### 2.2 Selection criteria employed

Articles that were selected by the research team were agreed upon by the team members that assessed the following criteria for inclusion: an existing theoretical or conceptual patient/person‐centred care framework; importance to patients (as validated by Zelinsky); frequently cited (as verified in Google Scholar); and provides interesting discussion or presents concepts important to patients that tend to be missing from the academic literature, which would allow for a comprehensive perspective in developing the PCC framework. A number of sources were excluded as the research team deemed saturation for developing domains and concepts. Due to the inclusive scope of the review and high variation among sources, critical appraisal was not conducted.

### 2.3 Synthesis

Common domains identified from the literature were reviewed, from which comparable themes and concepts were synthesized and then classified according to the Donabedian model for health care improvement into “Structure,” “Process” and “Outcomes.”^28^, ^41^ Among these three domains, each component is influenced by the previous and each is interdependent on the other.^41^ Secondly, the research team engaged in a series of facilitated discussions to develop and refine the framework, including parsing and combining domains, subdomains and components, which also helped the research team to determine the point of saturation with respect to domains and components, and cease further search in the literature.

## 3 DISCUSSION

### 3.1 Framework components

**Figure 1:**
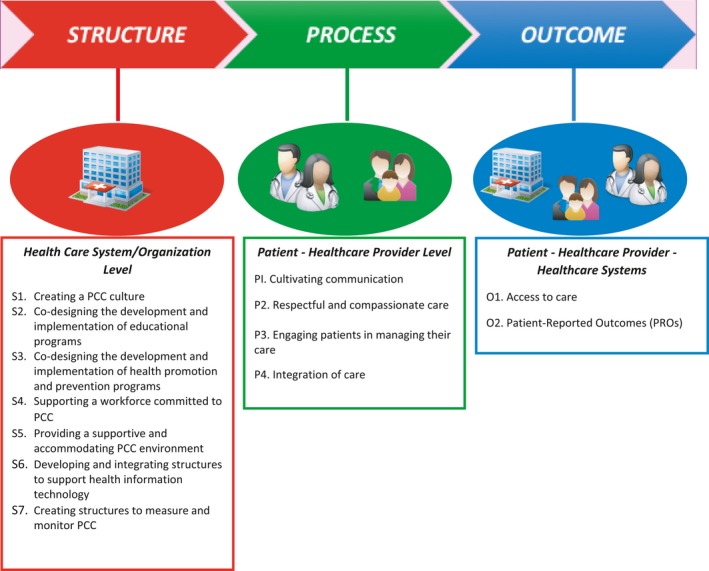
Framework for person‐centred care

Figure 1 presents the conceptual framework for implementing PCC. Structure includes PCC domains related to the health‐care system or the context in which care is delivered and provides the foundation for PCC – the necessary materials, health‐care resources and organizational characteristics. Process includes domains associated with the interaction between patients and health‐care providers. Outcomes show the value of implementing the PCC model, with domains relating to the results from the interaction between the health‐care system, HCPs and patients. The framework is organized like a roadmap, depicting the practical PCC implementation in the order that should be implemented – starting from structural domains that are needed as pre‐requisites, to facilitate processes and influence outcomes needed to achieve PCC.

### 3.2 Structure

Table 1 shows the seven core structural domains that have been identified as foundational components or pre‐requisites to promote a PCC model. The literature widely recognizes the importance of creating a PCC culture across the continuum of care (S1), where governments^42^ and organizations play a key role in the development of clear and comprehensive polices, processes and structures necessary for health‐care systems and health‐care providers to deliver PCC.^5^, ^30^, ^43^, ^44^ A common set of core values among all parties, as part of a strategic vision (S1a) is essential in the provision and receiving of care that includes patients, health‐care providers, communities and organizations within and outside of traditional health‐care services. While it is agreed that a key guiding principle in implementing PCC is to incorporate the patient perspective,^5^ there is a need to ensure that care is also patient‐directed, whereby patients are provided with sufficient and appropriate information to make decisions about their care and level of engagement.^5^, ^45^ Further, PCC respects individual patient beliefs and values and promotes dignity and antidiscriminatory care.^46^, ^47^ There is a need to be explicit in ensuring that diversity, including race, ethnicity, gender, sexual identity, religion, age, socio‐economic status and disability, is addressed and incorporated.^48^ A “rights‐approach” to PCC is aligned with the promotion of human dignity for both patients and health‐care providers and allows both parties to be aware of their rights and responsibilities.^14^, ^49^ Moreover, best practices demonstrate the need to standardize PCC language among patients, health‐care providers, policy makers, along with other key stakeholders to effectively foster a PCC culture of care (S1b).^5^, ^31^, ^44^ If the focus is in providing high quality of care, the terminology used by health‐care systems must change; PCC promotes the value of co‐design where health‐care providers do things *with* people, rather than “to” or “for” them.^13^

**Table 1:** Structure domains and components

Domain	Subdomain	Components	Sources
S1. Creating a PCC culture	S1a. Core values and Philosophy of the organization	Vision, Mission Patient‐directed: integrating patient experience and expertise Addressing and incorporating diversity in care, health promotion and patient engagement Patient and health‐care provider rights	5, 14, 30, 31, 45, 46, 47, 48, 49, 73
	S1b. Establishing operational definition of PCC	Consistent operational definitions Common language around PCC	5, 13, 31, 44
S2. Co‐designing the development and implementation of educational programs	Standardized PCC training in all health‐care professional programs	Integration of all health‐care sectors and professionals Professional education and accrediting bodies Translating into practice through continued professional education and mentorship	5, 24, 42, 49
S3. Co‐designing the development and implementation of health promotion and prevention programs	S3a. Collaboration and empowerment of patients, communities and organizations in design of programs	Identify resources Creating partnerships with community organizations Create patient advisory groups	5, 23, 43, 51, 52, 53, 54, 55
S4. Supporting a workforce committed to PCC	S4a. Ensure resources for staff to practice PCC	Provide adequate incentives in payment programs; celebrate small wins and victories Encourage teamwork and teambuilding	5, 43, 48, 56
S5. Providing a supportive and accommodating PCC environment	S5a**.** Designing health‐care facilities and services promoting PCC	Collaborate with and empower patients and staff in designing health‐care facilities Environments that are welcoming, comfortable and respectful Spaces that provide privacy Spiritual and religious spaces Facility that prioritize the safety and security of its patients and staff Areas/rooms that will support the accommodation of patients	5, 24, 29, 43, 57, 59
	S5b. Integrating organization‐wide services promoting PCC	Provide interpretation and language services Patient‐directed visiting hours	24
S6. Developing and integrating structures to support health information technology	Common e‐health platform for health information exchange across providers and patients	Electronic Health Record systems with capacity to coordinate and share health‐care interactions across the continuum of care Health information privacy and security E‐health adoption support through strategic funding and education	5, 43
S7. Creating structures to measure and monitor PCC performance	Co‐design and develop framework for measurement, monitoring and evaluation	Co‐design and development of innovative programs to collect patients and caregiver experiences about care received and providing timely feedback to improve the quality of health care (including complaints and compliments, wins and lessons learned) Reporting and feedback for accountability and to improve quality of health care	30, 43, 60, 61, 62, 63, 64, 65, 66, 67, 68, 69, 70, 71, 72

The lack of emphasis on PCC in medical education remains a barrier to its implementation,^5^ resulting in practices gaps. Specifically, current education tends to focus on the biomedical model, is not standardized across health‐care sectors and professionals, and is not co‐developed with patients and health‐care providers, (S2) despite successful models that incorporate both perspectives in the development and implementation of training.^43^ With the rapid emergence and evolution of PCC, there is a need for innovative education programs that are endorsed by key stakeholders and champions in medical education, including medical faculty, deans, administrative directors and accrediting bodies.^43^, ^50^ Educational programs should also include administrative staff, volunteers and allied professionals involved in health care, who are needed to support the cultural change.^24^ As integrating PCC into the health‐care curriculum does not necessarily translate into practice, PCC education programs should be designed to continue through informal training, continued leadership development and training through mentors and role‐models, eventually leading to a greater impact on culture change.^5^, ^24^

Patients and communities can also play a key role in co‐designing the development and implementation of health promotion and prevention programs (S3). By collaborating with and empowering patients, patient advisory groups,^5^, ^43^ communities and organizations, health‐care systems will be able to develop appropriate programs that meet the needs of all people.^51^, ^52^, ^53^, ^54^, ^55^ Building capacity of communities and organizations can also enhance integration, coordination and continuity of care, by supporting patients, and identifying resources that address barriers to accessing care and determinants of health (e.g housing, nutrition, education, etc.).^24^

Another major structural component is providing a supportive PCC work environment that ensures adequate resources for staff to practice PCC (S4). Current reimbursement models are one of the main obstacles for promoting and practicing PCC. Physician reimbursement is not typically linked to the importance placed on building and maintaining relationships and level of care quality provided as perceived by patients. Most current primary care payment systems encourage physicians to increase the number of patients seen and reduce time spent with individual patients.^56^ Policy makers need to consider alternative provider payment methods and incentives to reward practicing PCC.^5^, ^43^, ^56^ Additionally, to promote a supportive PCC work environment, Epstein et al suggest creating “communities of care,” which work to promote teamwork, collaboration and communication among HCPs to collectively meet the needs of their patients.^48^

A supportive and accommodating built environment is an essential aspect of PCC (S5) where co‐design with patients is crucial to ensure that patients feel comfortable, welcomed and have their needs met.^5^, ^24^, ^28^, ^43^ Healing environments that support choice, dignity and respect have a positive impact on health‐care outcomes (S5a). The physical design of the health‐care environment influences patient safety (reducing errors, patient falls, infections, etc.) and patient experience (supporting privacy and comfort).^57^, ^58^ Further, environments should integrate services to accommodate patients, such as language support and, appropriate and flexible visiting hours.^24^ Several well‐established patient‐centred organizations (i.e Planetree^59^) provide consultation services to HCPs to develop PCC environments and support implementation.

Developing a common e‐health platform for health information exchange across providers and patients with the capacity to link all health‐care electronic data across the continuum of care must also be implemented (S6). Such structures include Electronic Medical Records, which have proven to support access, coordination and safety in care delivery, through enhancing health‐care processes (information access, patient‐health‐care provider communication, patient and family involvement, etc.). E‐health technologies should provide secure and private platforms and its integration involves both building and updating existing health‐care facilities, and effectively connecting patients and caregivers with practitioners throughout the continuum of care.^5^, ^43^

Finally, patients, health‐care providers and policy makers should co‐develop structures to measure and monitor PCC performance based on feedback from patients, to promote PCC practice (S7). Measurement approaches include the use of patient experience surveys, patient‐reported outcome measures in clinical care, patient complaints and complements, alongside reported wins and lessons learned.^30^, ^60^, ^61^, ^62^, ^63^, ^64^, ^65^, ^66^, ^67^, ^68^, ^69^, ^70^, ^71^, ^72^ Utilizing existing public reporting systems present an ideal platform for PCC measuring, reporting and providing accountability.^43^ Feedback should also be tailored to the audience. For instance, while patients may be concerned with access to care and relationships with health‐care providers, policy makers may utilize the information in assessing health‐care utilization and costs. Health‐care systems are developing innovative programs to collect data from patients and report this information back to patients and health‐care providers in an accurate and timely manner during clinical encounters to support patient self‐management via visual dashboards.^63^

In implementing these structural components, the balance between health‐care providers and patient burden and prioritization of issues must be acknowledged. In addition, quality improvement leaders need to be included in the development of these programs.^6^ Having a clear vision on how PCC strategies fit within overall health‐care system, quality improvement is critical in improving PCC processes and outcomes.^5^

### 3.3 Process

Four process domains were identified, each of which builds upon the last during a patient‐health‐care providers interaction (Table 2). Beginning with cultivating communication (P1), evidence has shown that when a patient's values, needs and preferences are incorporated into health‐care practice, communication better enables patients to be active participants in their own care.^73^, ^74^, ^75^, ^76^, ^77^, ^78^, ^79^, ^80^ Positive associations between physician communication skills have been associated with positive patient outcomes such as increased patient satisfaction, recall, understanding and adherence to therapy.^35^, ^81^, ^82^ Components of communication include listening to patients *(*i.e gathering information through active listening and seeking patient's informational needs) (P1a.), sharing information (P1b.) and discussing care plans with patients (P1c). When combined, this would facilitate PCC and enhance patient care. Enabling physician competency in practicing person‐centred communication through teaching has been shown to be an effective way to implement this style of communication.^83^ Techniques such as using open‐ended questions to invite patients to reflect on their condition, pain, symptoms and other areas of life that may be linked to this, and eliciting the patient's reactions to the information given should be practiced to initiate and continue engaging in PCC dialogue.

**Table 2:** Process domains and components

Domain	Subdomain	Components	Sources
P1. Cultivating communication	P1a. Listening to patients	Gathering information through active listening Asking questions of what patients want to discuss (concerns, views, understanding) Non‐verbal behaviours (eye‐contact, listening attentively, proximity/touch, head nodding)	20, 73, 74, 75, 82
	P1b. Sharing information	Patients are provided with all the necessary information to make informed decisions in relation to their diagnosis and treatment plan Sharing of information regarding patient's condition and their own impact/influences on their condition	20, 31, 75, 82
	P1c. Discussing care plans with patients	Responding to patient and caregiver needs Aim and follow‐up of treatment or interventions with possible outcomes and adverse events/side‐effects Discussing and building capacity of patients for self‐management and self‐care Acknowledging and discussing uncertainties Creating a shared understanding	20, 80, 81
P2. Respectful and compassionate care	P2a. Being responsive to preferences, needs and values	Acknowledge the patient as an expert in their own health and as a part of the health‐care team Understanding patient within his/her unique psychosocial or cultural context (i.e: awareness of religious, spiritual, lifestyle, social and environmental factors) Responding empathically	20, 28, 31, 78, 82
	P2b. Providing supportive care	Building a partnership with patients Providing resources Sensitivity to emotional/psychosocial needs	31, 78, 79
P3. Engaging patients in managing their care	Co‐designing care plans with patients	Shared decision making Goal‐setting Supporting self‐care management Care plans can be accessed by patients and health‐care providers	80, 91
P4. Integration of care	Communication and information sharing for coordination and continuity of care across the continuum of care	Between health‐care providers Referrals to specialist Discharge communication Providing access to information and resources	79, 93, 95, 96, 107, 108, 109, 110

With effective communication comes the provision of respectful and compassionate care (P2). This includes being responsive to patient preferences, needs and values^20^ through acknowledging the patient's personal, cultural, religious and spiritual values, while expressing empathy, sympathy and reassurance, and responding to the patient's emotions.^82^ Providing respectful care fosters relationship building and has been shown to promote healing and better outcomes.^20^ To provide respectful and compassionate care, one must acknowledge the patient as an expert in their own health, and through this, develop partnerships that allow for sensitivity to emotional and psychological needs and empathetic responses.^31^ It has been shown that compassion decreases in the latter years of medical training, in which by the time the health‐care providers completes their training, they have become more desensitized to empathic processing.^84^ Compassion‐cultivation programs, including mindfulness implemented throughout medical training, have been shown to have effective long‐lasting results.^85^

Engagement of patients with health‐care providers is important (P3), as it effectively influences both the overall health‐care experience, but also improves health‐care provision^27^, ^86^ both patients and health‐care providers feel respected, listened to and empowered. When doctors and nurses are engaged with their patients, they are less likely to make mistakes.^87^, ^88^, ^89^ Engagement includes co‐designing care plans, which includes aspects of shared decision making, goal‐setting and support, all of which assist clinical management and contribute to better health outcomes, improved quality of care^90^, ^91^ and improved patient safety.^92^

The fourth process domain of integration of care (P4) is based on the fragmentation of nationally funded health‐care services found in many countries where there is insufficient or complete lack of communication of patient information and linkage of health services between different health‐care providers, such as between acute and community health‐care providers and with the private sector.^93^ Components that can support and link disjointed services and information across the continuum of care include new e‐health technologies,^94^ with platforms being developed to enable interactions with health‐care providers for continuity of care.^95^, ^96^ For instance, improved accessibility to medical information through electronic patient portals (patients have direct and private access to their medical records), and the use of email communications between patients and health‐care providers.^95^, ^96^

### 3.4 Outcomes

Outcomes derived from PCC need to be real and tangible, to show the value of implementing this type of model. Four outcome domains were identified (Table 3). Access to care (O1) is defined as the system's capacity to provide care efficiently after a need is recognized, as well as costs associated with receiving care.^97^ A person‐centred access model acknowledges the structures that may result in physical or financial barriers, as well as or other determinants of health‐care access;^97^ it can help patients secure appropriate and preferred health care at the right time to promote improved health outcomes while reducing costs to the health‐care system.^84^, ^98^ A Timely access to care is often cited as an outcome of PCC (O1a.), which is not only wait times for operations and referrals, or the time needed during a consultation or waiting for test results, but also the availability of health‐care providers during and outside working hours (O1b.). Improving timely access to care has the potential to reduce hospital admissions, decrease utilization of health‐care services (e.g emergency department visits and hospital length of stay) and also may help to reduce morbidity and mortality for both acute and chronic disease.^99^, ^100^ Opportunities for cost savings that are relevant to both patients and the system must also be identified, particularly the lack of affordability of health‐care services, which can have a negative impact on patients and families.^97^ For instance, ambulance and emergency care, and pharmaceutical costs can hinder access to care, jeopardizing patient safety. These considerations should be incorporated into the PCC finance model and assessed as part of the costs of care by the health‐care system.^97^ Although PCC provision will have an initial price tag, in the long run, supporting patients to make their own decisions about their care establishes better value for money, ensuring that the expenditure and cost attached to PCC implementation goes towards what patients value most. The UK Health Foundation report – “Person‐centred care made simple” presents evidence about cost savings and reductions in health‐care services utilization,^42^ that is when people are better informed, they may choose different treatments – often those that are less invasive and less expensive when people are supported to manage their own care more effectively,^101^, ^102^ are less likely to use emergency hospital services.^90^

**Table 3:** Outcome domains and components

Domain	Subdomain	Components	Sources
O1. Access to care	O1a. Timely access to care	Wait times for referrals to see specialists, to receive a consult During consult, to be seen at emergency community care, pre‐hospital, hospital, post‐hospital; secondary care; time for patient care	83, 98, 99
	O1b. Care availability	Availability of health‐care practitioners during and outside of working hours	83, 97, 98, 99
	O1c. Financial burden	Affordability of care including complimentary care and therapies, dental, pharmacare, ambulance	97
O2. Patient‐Reported Outcomes (PROs)	O2a. Patient‐Reported Outcomes Measures (PROMs)	Health‐Related Quality of Life Symptoms Functionality Psychosocial outcomes	65, 67, 72, 112
	O2b. Patient‐Reported Experiences (PREMs)	Recommendation or rating of hospital, health‐care provider Assessment of care, including appropriateness and acceptability of care (competency, knowledge, skills of staff)	58, 71, 112
	O2c.Patient‐Reported Adverse Outcomes (PRAOs)	New or worsening symptoms Unanticipated visits to health‐care facilities Death	105

The impact of PCC on outcomes can be informed through the use of Patient‐Reported Outcomes (PROs). PROs are patient‐centric measures comprised of information from patients about a health condition and its management,^72^ providing a connection between health‐care service provision and outcomes (O2). For instance, Stewart et al highlighted that once patients perceived the visit to be person‐centered, they experienced better recovery and emotional health, and fewer diagnostic tests and referrals two months later.^51^ De Silva described how when people are supported to manage their own care more effectively, they are less likely to use emergency hospital services.^103^ Bertakis et al reported that patients who received a higher average PCC practice style during clinic visits had lower odds of using specialty clinics.^104^ Specific PROs that may be implemented include Patient‐Reported Outcome Measures (PROMs; O2a.), which measure patient health status, quality of life and symptoms, functionality, physical, mental or social health;^67^ Patient Reported Experience Measures (PREMs; O2b.) measure patient experiences with the health‐care system;^58^ and Patient‐Reported Adverse Outcomes (PRAOS; O2c.).^105^ Integrating these measures into clinical practice have shown to improve outcomes as well as improve quality of care.^58^, ^64^, ^68^

### 3.5 Study limitations

A limitation of our study is not conducting a critical appraisal of the sources used. However, it was agreed by the study team that the more inclusive approach to our text selection – including a variation in sources, both from the academic and grey literature (including government documents and patient organizations), it would not be appropriate to assess the quality of the sources in a systematic way. Further, as we were looking to obtain a comprehensive perspective to PCC and were most interested in concepts that were identified as important to patients, but tended to be missing from much of the peer‐reviewed literature. For example, concepts such as those pertaining to the structural domain tended to be more salient in the grey literature, which is not easily assessed for quality. Moreover, other limitations include the inclusion of only English texts. Additionally, we attribute much of the strength of this framework in being informed by the patient experience and supported by evidence, there is a need to validate the framework with additional patient perspectives to ensure that the concepts reflect what really matter to patients.

### 3.6 Strengths of the framework and applicability

The newly developed evidence‐based, patient‐informed framework captures key factors to comprise a PCC model, including best practices identified by various organizations that ensure the patient perspective is reflected alongside health‐care providers and the system. Using the Donabedian health quality improvement model to classify PCC domains, this framework provides a roadmap to guide the implementation of a PCC model.

There is no doubt that health‐care systems are interested in moving towards a more person‐centred‐based approach as systems rethink the way health‐care is provided and the role patients and families play in it. In the last decade, the popularity of PCC has led to the development of several frameworks^5^, ^9^, ^11^, ^20^, ^24^, ^28^, ^29^, ^30^, ^31^, ^32^, ^33^, ^34^, ^35^, ^36^, ^37^, ^38^, ^106^ by academia and quality improvement agencies; however, there are important gaps that are addressed in this framework. Where other frameworks have lacked comprehensive structure‐level implementation guidance, this framework provides an in‐depth discussion on the structural pre‐requisites that support the establishment of a PCC model which allow processes and outcomes to transpire. For instance, embracing and promoting a PCC culture is essential in laying the foundations for PCC; without a culture that genuinely values the perspectives of patients, alongside health‐care providers and managers, there will be little impetus towards developing effective education, programs and systems that will help to foster PCC.

Further, this framework illustrates an integrated PCC delivery system that can be operationalized at a systems level. Scholl et al^11^ proposed an integrated model of PCC that encourages inter‐professional interactions with patients and introduces new measures and interventions to inform the development of health‐care policies that could ultimately support the shift towards PCC. There are an array of models available that are designed according to the characteristics of the jurisdictions they serve.^107^ The integrated models of care concept are based on the coordination of services and abolition of care silos, specifically, the Planetree model of care, is an example of an integrated and PCC model.^107^, ^108^, ^109^, ^110^, ^111^ A reduction in fragmentation and enhancement in integration can result in more efficient services and supports the delivery of care across the continuum of care.

Finally, our proposed framework provides a unique perspective: incorporating international viewpoints, concepts and literature, as well as integrating best practice and patient perspectives, while focusing on implementation and quality improvement. In this way, the framework is highly generalizable and can be adapted to multiple health‐care contexts. It is important to note that while this framework can provide guidance on implementation, it is not meant to be a “blanket approach” to PCC; there is still a need for health‐care systems to be responsive to their specific contexts and identified priorities, while encouraging innovation for PCC.

### 3.7 Future research

As discussed in our study limitations, there is a need to validate the framework with additional patient perspectives. Future research involves conducting a qualitative study with diverse individuals and communities on “what matters to them,” and mapping these concepts to the framework to validate the concepts or identify any revisions or additions needed.

Additionally, while this framework focuses on implementation of PCC, there is still a need to incorporate person‐centredness into the measurement of health‐care system performance. Despite substantial efforts by health‐care systems and organizations to develop measures, such as PROs,^112^ the WHO has acknowledged that “as of yet, there are no universally accepted indicators to measure progress in establishing integrated people‐centred health services.”^6^ To assess how health‐care systems are implementing PCC, systematic measurement, the use of person‐centric indicators that monitor and evaluate PCC can help to guide quality improvement within organizations, as well as keep them accountable through public reporting.^113^, ^114^ To address this gap in standardized PCC measurement, we intend to use the framework domains to help us classify, identify and develop potential PCC measures and quality indicators that could be implemented at the system level.

## 4 CONCLUSION

In summary, this framework provides a step‐wise roadmap for health‐care systems striving to implement PCC. While people can relate to the PCC concept, health‐care providers and policy makers must embark towards this cultural shift in practice, and systems must be willing to adopt and create innovative models that are conducive to providing incentives to support and practice PCC. The adoption of PCC comes with challenges, and entails critical changes, particularly with regard to how care delivered and how patients and their providers interact. However, despite the challenges associated with this shift, the benefits of PCC are evident presenting a major opportunity for improving health outcomes; PCC is our future. To improve health and health care, health‐care systems must find a way to effectively implement and measure PCC.

## CONFLICT OF INTEREST

The authors declare no conflict of interests.

## AUTHORS’ CONTRIBUTIONS

MJS conceived the study, and all authors identified key literature to be included in the review. All of the authors participated in the development and refinement of the framework, including discussions and drawing on the patient and public experience and perspective, with leadership from SZ. RJJ and KM managed the references and design of the figures and tables. MJS led the drafting of the manuscript and key discussion points, with subsequent versions modified and manuscript preparation by KM, RJJ and HQ provided important intellectual contribution, guidance throughout the development of the manuscript and final revisions and guidance from ML on the presentation of the findings. All of the authors contributed to critical review and revisions to the manuscript, agreeing on the final version.
